# The Defects of Medical Assurance

**Published:** 1894-05-05

**Authors:** 


### The Institutional Workshop.

### HOSPITAL FINANCE.

### VII.—THE DEFECTS OF MEDICAL ASSURANCE.

In a previous article it was urged that the receipt of good wages cannot be held to disqualify a man in sickness from accepting the benefits offered by hospitals. In-patients do, in fact, consist largely of this class. They do not want to be entirely dependent on the charitable public for their treatment. Many of them suffer keenly under the loss of their independence. But what can they do? Home nursing is in the majority of cases an impossibility, and past savings are heavily taxed to meet the stoppage of wages, even where some allowance from club or benefit society is available for the needs of the family.

The class only a little removed from those who refuse to receive free medical aid are often placed at a very cruel disadvantage, and are worthy of more consideration than they receive. The small shopkeeper, the clerk, the teacher, the countless multitudes of minor professional people, who struggle desperately all their lives without ever attaining the haven of a margin to their incomes; these are among some of the hardest working members of the community, and for them sickness means too often a hopeless battle with exhausted resources wholly unaided by the skill and care which are within immediate reach of the very poor, or the frankly improvident.

It is certain that all these, and indeed all regular wage-earners could, while in health, contribute a weekly or monthly sum towards the expenses of the inevitable hour of sickness. But the one set are not allowed, the other not encouraged to make this necessary provision.

Sick assurance is no new theory. It forms a leading feature in every benefit society, and is practised in connection with many provident dispensaries. But as hitherto organised, two grave defects tend very seriously to cripple its usefulness and bar its spread.

In the first place, it seldom succeeds in securing for its subscribers skilled attendance at all equal in value to what can be obtained gratis. Thus the subscriber obtains no advantage beyond the satisfaction of his honest independence. His next-door neighbour, with precisely the same income, can secure for his child, without payment or difficulty, higher skill and infinitely more zeal than the average club doctor brings to his doctor. This is no hasty supposition. It is a well ascertained fact, and accounts in no small degree for the unparalleled abuse of medical charities in the present day. The man who has no club, and yet desires to be free from charitable assistance, is of course in rather worse case. The medical practitioners who work for pay among the working classes at fees varying from 6d. to 2s. a visit, are at a cruel disadvantage when called upon to compete with the network of free dispensaries and out-patients' departments for which free letters can be obtained for the asking through countless charitable agencies. Some of these men are a credit to their profession, and do more work for less pay than would readily be believed. Too often, however, they are men who have failed elsewhere; who dislike their work, and despise their patients; who grow yearly more careless in treatment and perfunctory in attendance, yet are accepted by the poor with a stoical submission that is at once pathetic and provoking. Of two independent men, the club member will probably be better doctored than the outsider, but neither will fare half so well as the third, who resigns himself without an effort to charitable aid.

But sick assurance has another and, under some aspects, an even graver defect. It excludes hospital treatment, or rather hospital maintenance, altogether form its sphere. True, the subscriber can readily obtain through his society a letter for any hospital he may wish to enter. But what this amounts to is simply this—that the society having entered into an agreement to provide the subscriber with medical attendance conveniently hands him on to an institution where he may receive this gratis, and thereby frees itself from the obligation. The actual monetary cost of the letter is hardly worth considering. It is quite time that those who purchase letters with a view to distributing them among persons having a definite claim upon them should give up all idea of benefiting the hospital by this transaction. A few examples will suffice to render this clear. St. Mary's Hospital furnishes to every subscriber of £3 3s. letters for three in and no less than eighteen out patients. The average cost of each in-patient at this hospital is £5; the average cost of each out-patient is roughly estimated at 1s. 6d. For the sum of three guineas, therefore, the so-called benefactor or club secretary obtains medical relief for his clients to the value of £16 7s. It is difficult to understand where the benefaction comes in. Again, the London Hospital offers three tickets per guinea, and more in proportion as the subscription increases. Assuming that two of these are for out-patients, the value of the relief offered for a guinea amounts to £5 3s. The North London Hospital for Consumption offers for three guineas to receive one in-patient, costing £10, and four out-patients, costing 3s. each. The only basis on which this arrangement could subsist is that most of the letters issued are not wanted by the subscriber, and will never be presented. But the custom of issuing what are practically cheques to bearer, representing a value of from three to five times the amount of cash paid down, can hardly be considered commercially sound, and lies open in practice to grave abuses. It is now becoming increasingly common for those who have no acquaintance among the poor to send all their letters to some charitable agency to distribute for them. This being the case, it might be desirable to issue a return to each subscriber stating the actual cost incurred by the hospital in response to the subscription. Large societies, such as the Saturday Fund, who use every letter to which they are entitled, would then be seen to constitute a heavy drain on the hospital resources. Even the Hospital Sunday Fund, which distributes letters to the amount of only half the contributions, does not go far enough in the right direction. It is not too much to expect that charitable agencies existing for the sole purpose of benefiting the hospitals should pay the full cost of each patient recommended by them. The *quid pro quo* which has been so much insisted on would not by this means be imperilled, but the *quid* would be pared down into some reasonable likeness to its co-relative.

The result of the present ruinous system is plainly seen in the never-ending burden of debt and difficulty which hangs round the neck of almost every hospital, and in many cases seriously impairs its usefulness, and the mischief is not only to the hospitals. Letters too lightly obtained are scattered broadcast among a people who are rendered by their means every year less independent and less scrupulous of resorting to public charity.

No system of sick assurance has yet approached this difficulty or made any attempt on behalf of its subscribers to pay a reasonable proportion of the expense of their hospital treatment, nor has any system yet been organised which has succeeded in placing the thrifty in possession of even equal advantages to those which are everywhere open to the recklessly improvident without question.

